# Assessing the risk of rapid radiographic progression in Hungarian rheumatoid arthritis patients

**DOI:** 10.1186/s12891-021-04192-x

**Published:** 2021-04-02

**Authors:** Edit Végh, János Gaál, Pál Géher, Edina Gömöri, Attila Kovács, László Kovács, Katalin Nagy, Edit Feketéné Posta, László Tamási, Edit Tóth, Eszter Varga, Andrea Domján, Zoltán Szekanecz, Gabriella Szűcs

**Affiliations:** 1 Department of Rheumatology, University of Debrecen, Faculty of Medicine, Nagyerdei str 98, Debrecen, 4032 Hungary; 2 Department of Rheumatology, University of Debrecen Kenézy Teaching Hospital, Debrecen, Hungary; 3 Department of Rheumatology and Immunology, Faculty of Medicine, Semmelweis University, Budapest, Hungary; 4 Department of Rheumatology, Pándy Hospital, Gyula, Hungary; 5 Department of Rheumatology, Aladár Petz Hospital, Győr, Hungary; 6 Department of Rheumatology, Hospital of State Railways, Szolnok, Hungary; 7 Semmelweis Hospital, Kiskunhalas, Hungary; 8 Department of Rheumatology and Immunology, University of Szeged, Faculty of Medicine, Szeged, Hungary; 9 Department of Rheumatology, Ferenc Markhot Hospital, Eger, Hungary; 10 Department of Rheumatology, András Jósa Hospital, Nyiregyháza, Hungary; 11 Department of Rheumatology, Borsod-Abaúj-Zemplén County Hospital and University Teaching Hospital, Miskolc, Hungary; 12 Department of Rheumatology, Ferenc Flór Hospital, Kistarcsa, Hungary; 13 Department of Rheumatology, Markusovszky Hospital, Szombathely, Hungary

**Keywords:** Rheumatoid arthritis, Anti-TNF therapy, Infliximab, Biological therapy, Outcome, Rapid radiographic progression

## Abstract

**Background:**

The outcome of rheumatoid arthritis (RA) should be determined early. Rapid radiological progression (RRP) is > or = 5 units increase according to the van der Heijde-Sharp score within a year. The risk of RRP can be estimated by a matrix model using non-radiographic indicators, such as C-reactive protein (CRP), rheumatoid factor (RF) and swollen joint count (SJC).

**Patients and methods:**

A non-interventional, cross-sectional, retrospective study was conducted in eleven Hungarian arthritis centres. We assessed RRP risk in biologic-naïve RA patients with the prevalence of high RRP risk as primary endpoint. RRP was calculated according to this matrix model. As a secondary endpoint, we compared RRP in methotrexate (MTX) responders vs non-responders.

**Results:**

We analyzed data from 1356 patients. Mean CRP was 17.7 mg/l, RF was 139.3 IU/ml, mean 28-joint disease activity score (DAS28) was 5.00 and mean SJC was 6.56. Altogether 18.2% of patients had high risk (≥40%) of RRP. RA patients with high RRP risk of RRP (*n* = 247) had significantly lower age compared to those with RRP < 40% (*n* = 1109). MTX non-response (OR: 16.84), male gender (OR: 1.67), erosions at baseline (OR: 1.50) and ACPA seropositivity (OR: 2.18) were independent predictors of high-risk RRP. Male gender (OR: 5.20), ACPA seropositivity (OR: 4.67) and erosions (OR: 7.98) were independent predictors of high RRP risk in MTX responders.

**Conclusions:**

In this Hungarian study, high RRP risk occurred in 18% of RA patients. These patients differ from others in various parameters. RRP was associated with non-response to MTX.

## Key points

- In Hungary, one-fifth of RA patients has rapid radiographic progression.
- MTX non-response, male gender and ACPA positivity were independent predictors of high-risk rapid radiographic progression.

## Introduction

Radiographic damage may be one of the most important outcomes of rheumatoid arthritis (RA). Therefore, we should identify patients at high risk for rapid radiographic progression (RRP) early, which should influence our treatment strategy. In such patients, effective therapy may reduce the odds of progression [1, 2]. Early, intensive treatment may slow down the rate of radiographic progression [1–3]. Various clinical and biological markers have been identified as baseline risk factors for radiographic progression. Optimally, the combination of multiple markers may improve the value of prediction [3]. Recent recommendations for the management of rheumatoid arthritis (eg. EULAR, Hungarian National Guideline) [1, 2, 4] introduce the importance of prognostic markers in treatment decisions in RA referring to the matrix risk model developed by Vastesaeger et al. [3]. These recommendations also suggest the early introduction of biologic therapy for patients with high risk of RRP [1, 2, 4].

In order to identify the need for earlier use of biologic treatment in the everyday clinical practice, it is crucial to estimate, which patients who would benefit the most from early aggressive therapy. The matrix risk model developed by Vastesaeger et al. [3] is an evidence-based, simple to use tool to assess the risk of RRP for patients with a specific combination of easily accessible variables. This model has been used in real-life on a community-based sample of patients with active RA naïve to biologic treatment. In a single center retrospective study in Hungary 100 RA patients were assessed [5]. Altogether 21% of consecutive patients with active RA had a high (≥40%) risk of RRP (vdHSS ≥5/year), and methotrexate (MTX) responsiveness was a key parameter in determining the RRP risk calculation [5]. Durnez et al. [6] validated a matrix model in their observational early RA cohort that was conceived based on data from the ASPIRE early RA trial. In this, as well as other studies, patients with longer duration of RA had lower RRP risk [3, 5, 7]. In addition, predictors of RRP based on other published matrix models are presence of anti-citrullinated protein antibodies (ACPA), baseline erosions and cigarette smoking [3, 5]. In the therapeutic guidelines the Hungarian Ministry of Health endorsed the use of the matrix prediction models in the therapeutic decisions regarding the initiation of biologic therapies [4]. Certainly, there have been other models that estimated RRP. For example, in various studies, 3-month DAS [8], anti-neutrophil cytoplasmic antibodies [9], various cartilage and proteoglycan turnover biomarkers [10], matrix metalloproteinases [11, 12], some genetic markers including tumor necrosis factor α (TNF-α) gene polymorphisms [11] have been suggested as predictors of RRP. Moreover, early MRI bone edema [13] and first-year radiographic progression determined further progression in early RA [14]. With respect to matrix-based risk models, Visser et al. [15] used autoantibodies, CRP, erosion score and treatment group in the BeSt study to determine RRP. This model was able to find differences in RRP in the four arms of the study [15]. Very recently, Vanier et al. [16] presented an updated matrix model by pooling individual data of DMARD-naïve active, early RA patients from numerous databases. Four parameters, such as RF positivity, presence of at least one erosion at baseline, CRP and SJC were retained [16]. On the other hand, there have been studies criticizing the applicability of these matrices in daily clinical practice. Lillegraven et al. [17] compared three of these models including two matrix-based models described above [3, 15]. This study, which also included biologic-treated patients, found that these models may have limited ability to predict RRP in early RA [17]. De Cock et al. [18] tested six matrices in 74 early RA patients with X-rays of hands and feet at baseline. They did not find these matrices fully reliable in RRP prediction in the daily practice [18].

Our objective was to determine the risk of RRP according to the matrix risk model developed by Vastesaeger et al. [3] among Hungarian RA patients as a joint effort of national arthritis centers. Although the original matrix model has been developed for the use in clinical studies to determine treatment efficacy [3], we wished to apply this model to select candidates for biological therapy among biologic-naïve patients. This was a non-interventional, cross-sectional, retrospective, population-based, nationwide survey based on hospital record data. This was a theoretical prediction as prospective follow-up of radiographic progression was not performed.

## Patients and methods

### Brief description of the matrix risk model

In the model of Vastesaeger et al. [3], RRP was defined as a threshold change in modified Sharp/van der Heijde score (SHS) of > or = 5 U/year. The developed and validated matrix risk model enables to determined RRP without actual radiographs based on three simple variables. In this model, the 28 swollen joint count (SJC), RF and CRP levels were used as trichotomous variables. These three variables weighed equally.

### Sample size calculation and patient selection process

We applied the precision-based sample size calculation with the following formula to calculate the minimum sample size: π (1-π)/e^2^, where *π* is the expected proportion, *e* is the required size of standard error. Precision is defined as the ± range around the estimated proportion, and was calculated as ±1.96 × *e* [19]. Calculating with π = 0.2 as the expected proportion and ± 0.02 as the required precision, the minimum required sample size was 1537. Assuming as the estimated proportion of patients with incomplete data will be 15%, the adjusted minimum sample size calculated was 1537/0.85, which equals 1808 patients. This was the number of patients required for the estimation with the specified precision of ±0.02 (data not shown).

A multi-stage sampling method was applied to ensure equal probability of selection of target patients. In order to obtain a population-based sample, each of the 20 regional rheumatology centers evenly distributed throughout Hungary (1st-stage sampling units) were invited to participate in the study. The number of cases per center (*n*) was allocated according to the number of treated RA patients in each center at the time of the start of the study. Investigators then collected data on patients up to the allocated sample size (*n*), thus ensuring sampling probability proportional to size of the 1st-stage sampling units (i.e., the 20 rheumatology centers).

Random selection of patients (2nd-stage sampling units) was then performed to avoid any selection bias. The list of all currently treated RA patients (sampling frame) was created in each center. The allocated number of patients was selected from the sampling frame with simple random sampling (with tables of random numbers) in order to each patient having the same chance of being chosen [20].

**Fig. 1:**
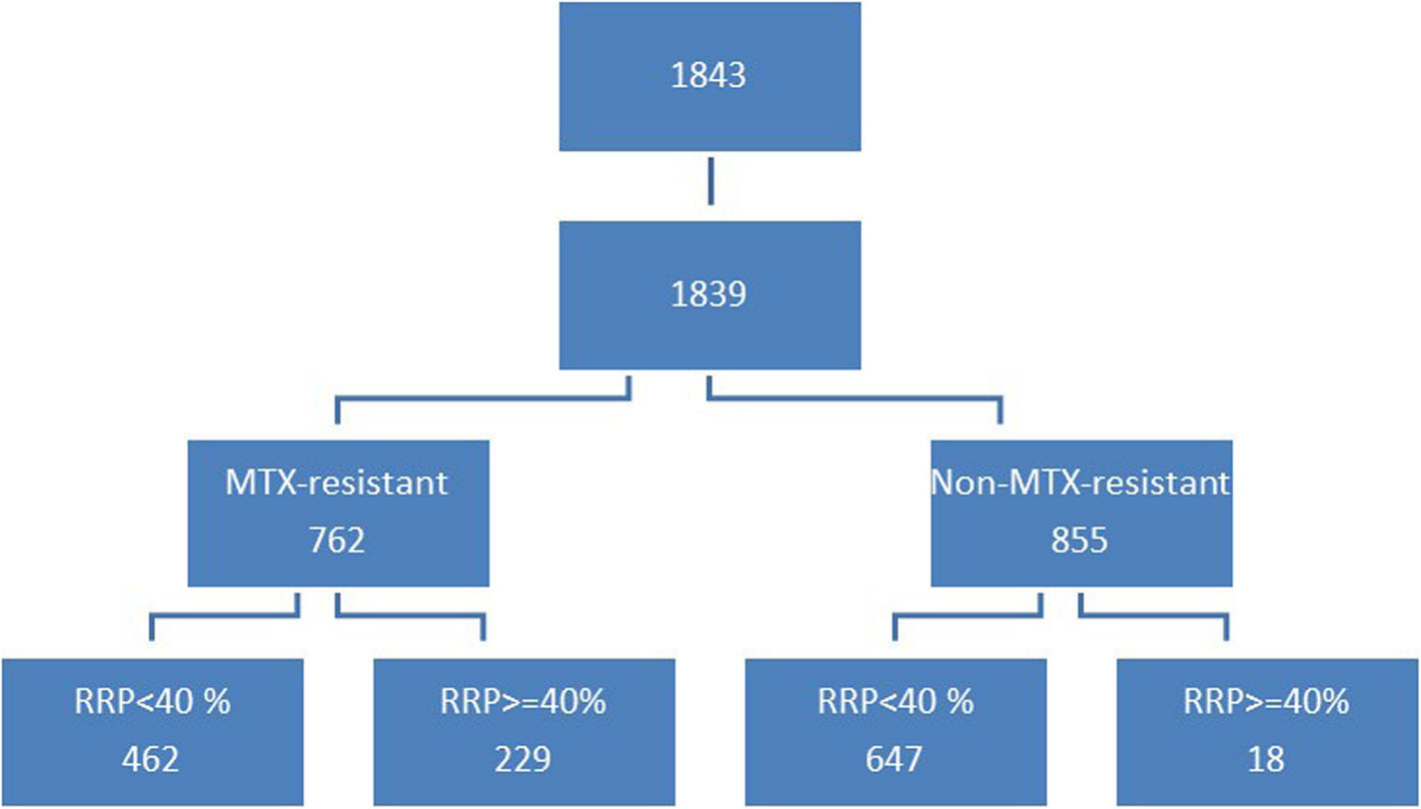
Flowchart of the study

Based on these calculations, 1843 patients were consecutively chosen for data analysis (Fig. 1). The only inclusion criterion was the diagnosis of RA. There were no exclusion criteria except for age ≤ 16 years (definition of juvenile arthritis).

### Data capture process

Patient data from the last visit that occurred right before the date of patient selection were collected. For those already on biologic therapy, the last data before initiation of biologics were retrospectively recorded. Thus, only data obtained from biologic-naïve patients were evaluated. We used clinical data obtained from hospital records and also assess radiographs at baseline for the presence or absence of erosions.

Standardized electronic spreadsheed (Microsoft Excel) was used to capture the data. The following data were collected based on hospital records:

- age
- gender
- duration of RA
- history of DMARD use (MTX and others; current/past/never use)
- MTX response (responder/non-responder)
- SJC, CRP and RF levels (for calculation of the matrix-based RRP risk)
- anti-citrullinated protein antibody (ACPA) status (positive/negative)
- DAS28 activity score
- presence of baseline erosions on radiographs at baseline (yes/no)
- cigarette smoking (current/past/never)

For determining RRP, we used the traditional three variables, SJC, RF and RF, as determined by Vastesaeger et al. [3]. However, we added a few binary variables described above in order to look for further denominators.

### Objectives

The primary objective was to estimate the prevalence of high (≥40%) risk of RRP in a community-based sample of RA patients, naïve to biologic treatment presenting at rheumatology departments. Active disease was defined as DAS28 ≥ 5.1, which is the threshold for the use of biologics in Hungary.

The secondary objectives were:

- to assess the difference in the prevalence of high RRP risk in RA patients classified MTX-non-responders versus MTX-responders (MTX non-responders are patients with DAS28 > 5.1 despite MTX treatment for at least 6 months in stable doses);
- to assess the multivariate association of patient characteristics with high RRP risk (independent of the parameters used in matrix model).

### Statistical analysis

The calculation of sample size is described above. MS Excel was used to record, summarize and clean the data. Statistical analyses were performed with IBM SPSS 20 program. Continuous variables were described by mean and standard deviation, the distribution was described with number of cases and percentage. Distribution was analyzed with Kolmogorov-Smirnov test. Between group difference was analyzed with Mann-Whitney test and Chi^2^-test. Independent predictive factors were identified applying univariable and multivariable regression analyses. We considered correlations to be significant in case of a *p*-value less than 0.05.

## Results

### Data collection

Initially 1843 consecutive subjects were recruited. Four subjects were excluded at the beginning due to age ≤ 16 years, 222 patients because of missing DAS28 scores and 261 patients due to missing data necessary for the calculation of RRP. Thus, in the end, data from 1356 RA patients could be included in the analysis. There were no missing data in these 1356 patients (Fig. 1). The demographic and clinical data of these patients are seen in Table 1. The mean age of the patients were 55.5 ± 13.3 years (range: 17–89) and the mean disease duration was 8.4 ± 8.8 years (range: 0–62). Altogether 1148 patients (85%) were women. MTX-non-responders were currently taking MTX + DAS28 > 5.1.

**Table 1:** Clinical and demographic data of the analyzed patients (*n* = 1356)

Age (years)	55.5 ± 13.3
Female gender (%)	85
Disease duration (years)	8.4 ± 8.8
RF positivity (%)	76
RF absolute level (IU/ml)	139.3 ± 196.4
ACPA positivity (%)	72
CRP level (mg/l)	17.7 ± 23.8
DAS28 score	5.00 ± 1.59
Swollen joint count (n)	6.56 ± 5.44
Current MTX therapy (%)	64
MTX therapy ever (%)	92
MTX non-responder (%)	51
current csDMARD therapy (other than MTX) (%)	35
csDMARD therapy ever (other than MTX) (%)	76
Presence of erosions (%)	61
Current smoker (%)	26
Risk of RRP (%)	26.8 ± 13.7
***Risk of RRP ≥ 40% (%) (primary endpoint)***	***18,2***

### Associations of various parameters with the risk RRP

First, the risk of RRP was calculated in all 1356 RA patients according to the matrix model [3]. The risk of 40% was the cutoff between high- and low-risk patients. Altogether 247 patients exerted RRP risk ≥40% (18.2%) and 1109 patients had low risk (81.8%) (Table 1).

**Fig. 2:**
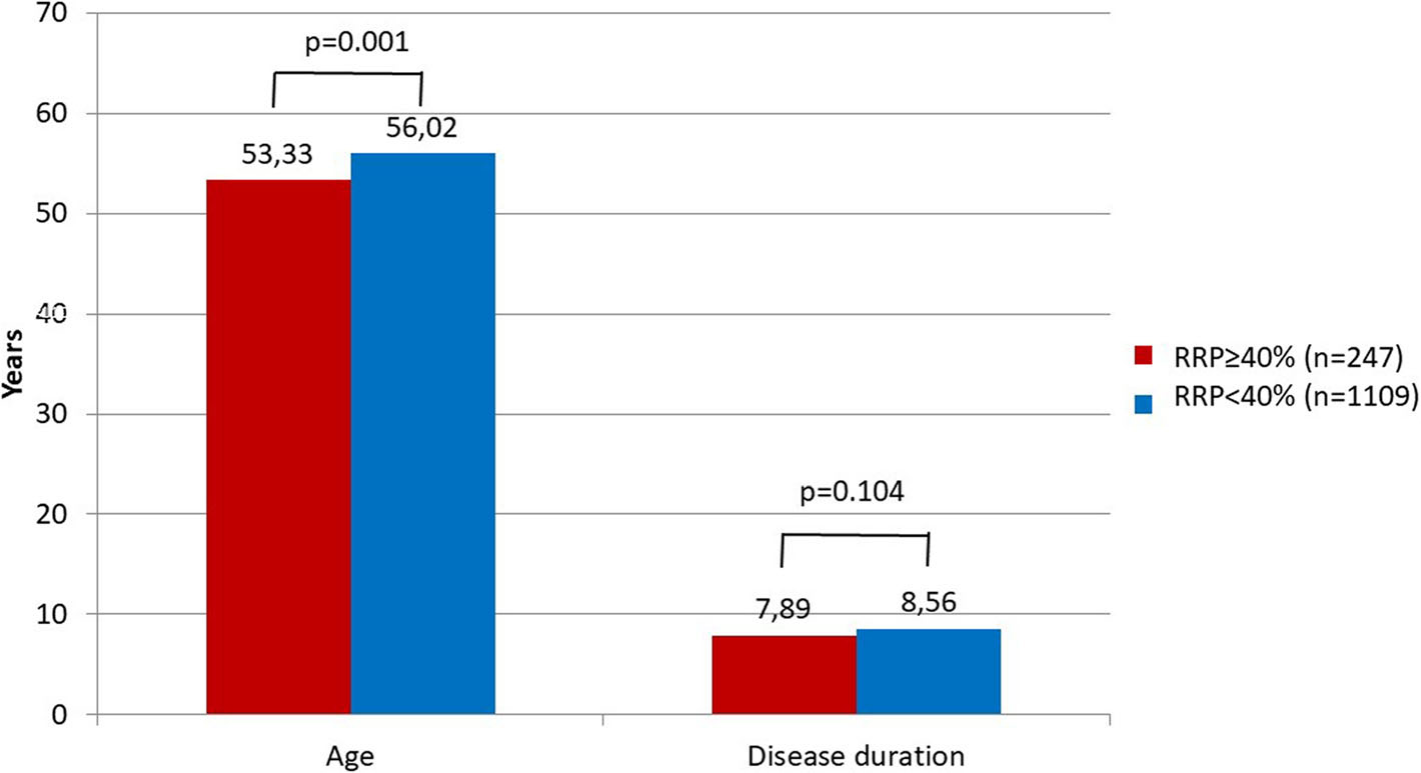
Associations of rapid radiographic progression (RRP ≥ 40%) with age and disease duration

Among continuous variables other than those used for calculation of RRP risk, RA patients with the risk of RRP ≥ 40% (*n* = 247) had significantly lower age than those with RRP < 40% (*n* = 1109) (53.33 ± 12.31 vs 56.02 ± 13.50 years; *p* = 0.001) (Fig. 2). These two patient groups did not show statistically significant difference in disease duration (7.89 ± 9.31 vs 8.56 ± 8.70 years, respectively; *p* = 0.104) (Fig. 2).

Binary variables included gender, ACPA status, baseline presence of erosions on radiographs, current smoking and MTX response status. With respect to binary variables, the risk of RRP ≥ 40% was significantly associated with non-response to MTX (OR: 17.82), male gender (OR: 1.53), ACPA positivity (OR: 2.11), the presence of erosions (OR: 1.37) and current smoking (OR: 1.66) (Table 2). Multivariable logistic regression analysis revealed that MTX non-response (OR: 16.84), male gender (OR: 1.67), erosions at baseline (OR: 1.50) and ACPA positivity (OR: 2.18) were independent predictors of high-risk RRP (≥40%) (Table 3).

**Table 2:** Association of binary variables with risk of RRP in RA patients

	RRP ≥ 40%	RRP < 40%	***p***	Odds Ratio	95% CI
**MTX-response (*n*** **= 1356):**					
**Non-responders**	229 (93%)	462 (42%)	***< 0.001***	17.820	10.87–29.21
**Responders**	18 (7%)	647 (58%)			
**Gender (*n*** **= 1356):**					
**male**	50 (20%)	158 (14%)	***0.018***	1.527	1.073–2.174
**female**	197 (80%)	951 (86%)			
**ACPA status (*n*** **= 1221):**					
**positive**	186 (83%)	697 (70%)	***< 0.001***	2.107	1.449–3.063
**negative**	38 (17%)	300 (30%)			
**Presence of erosions (*n*** **= 1204):**					
**yes**	159 (70%)	612 (63%)	***0.046***	1.371	1.004–1.870
**no**	69 (30%)	364 (37%)			
**Current smoking (*n*** **= 1034):**					
**yes**	70 (34%)	195 (24%)	***0.003***	1.656	1.191–2.303
**no**	137 (66%)	632 (76%)

**Table 3:** Independent predictive factors for high-risk (≥40%) of RRP

Patient population	Parameters	Odds Ratio	95% CI	***p***
**All patients (*n*** **= 1356)**	MTX non-response	16.843	9.348–30.350	***< 0.001***
	Male gender	1.673	1.030–2.717	***0.038***
	ACPA positivity	2.180	1.366–3.480	***0.001***
**MTX non-responders (*n*** **= 691)**	ACPA positivity	1.925	1.186–3.124	***0.008***
**MTX responders (*n*** **= 665)**	Male gender	5.202	1.658–16.323	***0.005***
	Presence of erosions	7.984	1.019–62.529	***0.048***

### Factors associated with non-response to MTX

As non-responsiveness to MTX may exert the far closest association with high risk of RRP, we performed a detailed analysis of factors significantly associated with MTX response.

Among the 1356 analysed cases, we identified 691 MTX non-responders (51%) according to the definition described above. As presented in Table 4, MTX non-responders exerted significantly lower age (*p* < 0.001), higher RF levels (*p* = 0.002), CRP levels (*p* < 0.001), DAS28 score (*p* < 0.001) and SJC (*p* < 0.001) than responders. Also more MTX non-responders had erosions (*p* = 0.033) and had been currently smoking (*p* = 0.03) at the time of the study as compared to responders. Finally, the mean risk of RRP was also significantly higher in MTX non-responders (37.8 ± 6.6%) compared to responders (15.3 ± 8.9%) (*p* < 0.001). On the other hand, the calculated risk of RRP was not different between MTX non-responders and responders. The risk of RRP was rather low in both patient subsets (6.5 ± 4.2% and 5.8 ± 2.0%, respectively) (Table 4).

**Table 4:** Comparison of clinical and demographic data in MTX non-responders vs responders (*n* = 1356)

Parameter	MTX non-responders (***n*** = 691)	MTX responders (***n*** = 665)	***p***
**Age (years)**	52.99 ± 12.15	58.16 ± 13.98	***< 0.001***
**Female gender (%)**	84	86	0.365
**Disease duration (years)**	8.17 ± 8.73	8.72 ± 8.92	0.292
**RF positivity (%)**	76	75	0.442
**RF absolute level (IU/ml)**	153.55 ± 203.52	123.89 ± 187.21	***0.002***
**ACPA positivity (%)**	72	70	0.139
**CRP level (mg/l)**	21.24 ± 25.73	14.00 ± 21.01	***< 0.001***
**DAS28 score**	5.99 ± 0.68	3.98 ± 1.61	***< 0.001***
**Swollen joint count (n)**	8.87 ± 4.98	4.01 ± 4.74	***< 0.001***
**Current MTX therapy (%)**	83	45	***< 0.001***
**MTX therapy ever (%)**	96	89	***< 0.001***
**current csDMARD therapy (other than MTX) (%)**	44	25	***< 0.001***
**csDMARD therapy ever (other than MTX) (%)**	82	71	***< 0.001***
**Presence of erosions (%)**	67	61	***0.033***
**Current smoker (%)**	29	23	***0.030***
**Risk of RRP (%)**	37.82 ± 6.64	15.25 ± 8.90	***< 0.001***
**Risk of RRP – if switched to (infliximab (%)**	6.54 ± 4.18	5.84 ± 2.00	0.408

MTX non-responsive and responsive patient subsets were also analysed separately with respect to the risk of RRP and associated factors. Among continuous variables, neither age nor disease duration was different between the RRP ≥ 40% and RRP < 40% subsets within the MTX non-responder group (data not shown). With respect to univariable regression analysis of binary parameters in MTX non-responders, high risk of RRP was significantly associated with ACPA positivity (OR: 1.96) and current smoking (OR: 1.56), but not with gender or the presence of erosions (Table 5). Multivariable logistic regression analysis revealed that ACPA positivity (OR: 1.925) was independent predictor of RRP risk ≥40% in the MTX non-responder subset (Table 3).

**Table 5:** Association of binary variables with risk of RRP in MTX non-responders (*n* = 691)

	RRP ≥ 40% (***n*** = 229)	RRP < 40% (***n*** = 462)	***p***	Odds Ratio	95% CI
**Gender**					
**male**	43 (16%)	69 (17%)	0.197	1.318	0.471–1.099
**female**	186 (84%)	393 (83%)			
**ACPA status:**					
**positive**	171 (82%)	307 (70%)	***0.001***	1.957	1.298–2.950
**negative**	37 (18%)	130 (30%)			
**Presence of erosions:**					
**yes**	145 (69%)	271 (66%)	0.485	1.135	0.795–1.619
**no**	66 (31%)	140 (34%)			
**Current smoking:**					
**yes**	66 (34%)	81 (25%)	***0.025***	1.558	1.055–2.302
**no**	126 (66%)	241 (75%)			

Similarly, in MTX responders (*n* = 665), neither age nor disease duration was different between the RRP ≥ 40% (*n* = 18) and RRP < 40% (*n* = 647) subsets (data not shown). Among the binary variables, high risk of RRP was significantly associated with male gender (OR: 3.98), but not with ACPA status, the presence of erosions or current smoking as determined by *p* values and confidence intervals (Table 6). However, multivariable logistic regression analysis confirmed that male gender (OR: 5.20), ACPA positivity (OR: 4.57) and the presence of erosions (OR: 7.98) were independent predictors of high RRP risk in the MTX responder subpopulation (Table 3).

**Table 6:** Association of binary variables with risk of RRP in MTX responders (*n* = 665)

	RRP > =40% (***n*** = 18)	RRP < 40% (***n*** = 647)	***p***	Odds Ratio	95% CI
**Gender**					
**male**	7 (40%)	89 (14%)	***0.009***	3.984	1.506–10.526
**female**	11 (60%)	558 (86%)			
**ACPA status:**					
**positive**	15 (94%)	390 (70%)	0.049	6.538	0.857–49.896
**negative**	1 (6%)	170 (30%)			
**Presence of erosions**					
**yes**	14 (82%)	341 (60%)	0.067	3.065	0.871–10.789
**no**	3 (18%)	224 (40%)			
**Current smoking:**					
**yes**	4 (27%)	114 (23%)	0.755	1.247	0.390–3.991
**no**	11 (73%)	391 (77%)			

## Discussion

This was the very first study that assessed the risk of RRP in a Hungarian multicentre cohort of RA patients in real-life setting. We found that 18.2% of RA patients exerted RRP risk ≥40% based on a model using 3 variables as input parameters. This proportion with high risk of radiographic deterioration and joint damage can be considered significant. In the analysed population, high risk of RRP was significantly associated with lower male gender, ACPA positivity, the presence of erosions and non-response to MTX. About half of the analysed patients were MTX non-responders. In MTX non-responders, RRP was significantly associated with ACPA positivity, while male gender, ACPA positivity and baseline erosions predicted RRP in MTX responders.

Ours was a biologic-naïve cohort of patients with established RA, which only theoretically assessed the risk of RRP not applying prospective evaluation of radiographic progression. There have been other similar studies using matrix-based prediction models, however, some of them had different study design. After the introduction of the model introduced by Vastesaeger et al. [3] also utilized by us, Durnez et al. [6] validated the same matrix in their observational cohort based on the ASPIRE early RA trial by also using radiographs. The model was useful to determine the predictive value of different treatment strategies in early RA. Visser et al. [15] applied a slightly different model by using autoantibodies, CRP, erosion score and treatment group as predictors in the BeSt study to determine RRP. This model included the evaluation of radiographs and was able to find differences in RRP between the four treatment strategies applied in the BeSt study [15]. Vanier et al. [16] have recently developed an updated matrix model, where, in addition to RF positivity, SJC and CRP, baseline erosions were also used as predictors. Data were pooled from numerous large early RA cohorts including registries and clinical trials. This model could determine RRP probability with high precision. Although the matrix had moderate sensitivity and specificity, the authors found it useful for daily practice [16]. In contrast, when Lillegraven et al. [17] compared three matrix-based models including the one applied in our present study, they found that these models may have limited predictive value for RRP [17]. However, that study, in contrast to ours, has been performed in patients with early and not established RA and the study of Lillegraven at al [17] also included patients already receiving biologics. De Cock et al. [18] did not find these matrices reliable in the daily clinical practice. Yet, the majority of studies, similarly to ours, found matrix-based predictive models useful to simply predict RRP in the clinic.

We analyzed the risk of RRP in context with the presence of absence of baseline erosions. It may be considered strange to use erosions to predict further radiographic progression, however, as mentioned above, others have also used baseline erosions in their predictive models [14, 16].

In conclusion, this is the first biologic-naïve Hungarian RA cohort that assessed the risk of RRP. In this study, high RRP risk was determined in 18% of the patients. These patients differ from others in various clinical and serological parameters. RRP has also been associated with non-response to MTX. Our data, together with other studies, suggest that such models may be useful to predict radiographic progression in the daily practice.

## Data Availability

The datasets used and/or analysed during the current study available from the corresponding author on reasonable request.
